# Nanocarriers for Nitric Oxide Delivery

**DOI:** 10.1155/2011/936438

**Published:** 2011-08-22

**Authors:** Juliana Saraiva, Samantha S. Marotta-Oliveira, Simone Aparecida Cicillini, Josimar de Oliveira Eloy, Juliana Maldonado Marchetti

**Affiliations:** Faculdade de Ciências Farmacêuticas de Ribeirão Preto, Universidade de São Paulo, 14010-903 Ribeirão Preto, SP, Brazil

## Abstract

Nitric oxide (NO) is a promising pharmaceutical agent that has vasodilative, antibacterial, and tumoricidal effects. To study the complex and wide-ranging roles of NO and to facilitate its therapeutic use, a great number of synthetic compounds (e.g., nitrosothiols, nitrosohydroxyamines, N-diazeniumdiolates, and nitrosyl metal complexes) have been developed to chemically stabilize and release NO in a controlled manner. Although NO is currently being exploited in many biomedical applications, its use is limited by several factors, including a short half-life, instability during storage, and potential toxicity. Additionally, efficient methods of both localized and systemic *in vivo* delivery and dose control are needed. One strategy for addressing these limitations and thus increasing the utility of NO donors is based on nanotechnology.

## 1. Introduction

### 1.1. Diverse NO Functions

Nitric oxide (NO) is a free-radical gas and one of the smallest endogenous molecules with the ability to function as a chemical messenger, particularly in cells of the vascular endothelium and immune and neural systems. NO plays a critical role in regulating a diverse range of physiological processes, including cellular differentiation and apoptosis [1–10]. 

Medical and scientific interest in NO has grown exponentially since 1992, when it was nominated “Molecule of the Year.” Its documented physiological impacts are ever-expanding [11]. Until 1987, NO was known solely as a dangerous atmospheric pollutant generated by industrial processes and automotive engines and as a potential carcinogen [12, 13]. However, by the end of 1987, the discovery of NO synthesis in mammalian cells revealed that this molecule exerts physiological effects, many of which still have not been completely characterized [8, 13]. This discovery led to a rapid increase in research focused on NO [14–22]. NO is now known as one of the most important mediators of intra- and extracellular processes and is a major target of the pharmaceutical industry [12].

Endogenous NO is produced enzymatically by three distinct nitric oxide synthases via L-arginine conversion. The NO generated by each enzyme differs considerably in its pattern of expression and regulation, likely reflecting site-specific functions [13, 23]. These functions result in both beneficial and detrimental outcomes [12]. Regarding the former, NO may help to improve the prognosis of different human pathologies, including cardiovascular, hematological, metabolic, gastrointestinal, respiratory, neurological, renal, genitourinary, musculoskeletal/connective tissue, and obstetric/gynecological diseases as well as cancer [13]. Some of the specific functions of NO are as follows.

- Maintenance of Vascular Tone and Blood Pressure: Vascular tone is usually maintained by a steady release of tiny amounts of NO from the vascular endothelium. This NO release is triggered by friction exerted by circulating cells (shear stress) and results in slight vasodilatation [13, 24]. Blood pressure and pulsate flow also regulate the release of NO under physiological conditions, with NO inhibition leading to a drastic increase in blood pressure [12, 25].
- Regulation of Immunity and Inflammation: NO is an important cytotoxic mediator of activated immune cells capable of killing pathogenic agents, such as bacteria, parasites, and viruses, as well as tumor cells. NO can also inhibit the inflammation of blood vessels by blocking exocytosis of various mediators from endothelial cells, macrophages, and cytotoxic T lymphocytes [12, 13].
- Inhibition of Monocyte and Neutrophil Adhesion to the Vascular Endothelium: NO donors have shown to be potent inhibitors of neutrophil and monocyte adhesion to the vascular endothelium, a complicating factor in the pathogenesis of atherosclerosis [12, 26].
- Antiproliferative Effects: Cellular proliferation in the muscular layer of the blood vessel has a key role in narrowing the vascular lumen. NO produced by the vascular endothelium or arising from exogenous donors can inhibit this proliferation although the mechanism underlying its antiproliferative activity is not well understood [12, 27, 28].
- Antioxidative Effects: Oxidative stress contributes to thromboembolic disease. NO induces the production of the enzyme superoxide dismutase in the muscular layer of the blood vessels and in the extracellular space, decreasing the _O2_– available and the production of ONOO– [12, 29].
- Regulation of Neurotransmission: NO regulates the activity of certain motor neurons in the parasympathetic branch of the autonomic nervous system [13].
- Regulation of Platelet Function: NO mediates the adhesion and aggregation of platelets [13].
- Direct and Indirect Stimulation of Endocrine and Exocrine Secretion: NO regulates the release of gonadotropin-releasing hormone (GSH) from the hypothalamus and adrenaline from the adrenal medulla as well as exocrine secretions (e.g., amylase from the pancreas) [13].
- Regulation of Kidney Function: Release of NO at the level of the glomerulus increases blood flow and the rate of filtration, and urine formation [13].
- Regulation of Reproductive Function: NO can improve penile erection, fertilization and uterine relaxation during pregnancy [13].
- Role As a Messenger/Modulator: NO functions in a variety of essential biological processes [12].

Meanwhile, in addition to its beneficial effects, NO is a potentially toxic agent. This toxicity is particularly apparent during oxidative stress; when NO generates, O_2_ intermediates and leads to antioxidant deficiency [12].

### 1.2. NO Donors and Potential Therapeutics

Research on the biological functions of NO and other reactive nitrogen species requires exogenous sources of NO donors, which may serve as both research tools and drugs. Since mid-1980, newly developed NO donors have offered several advantages over older donors, such as spontaneous NO release or controlled release targeting certain tissues. The synthesis of molecules capable of releasing optimal amounts of NO at the right time and the right place poses a great challenge to pharmaceutical research. Several known drugs have demonstrated partial or total modulation of NO metabolism with diverse therapeutic results. Classic organic nitrates particularly showed beneficial therapeutic effects, yet they can induce such undesirable effects as tolerability, abrupt cephalea, and hypotension [13].

The classification of NO donors can be confusing, because all have the potential to be oxidized or reduced, producing reactive nitrogen species. However, similar chemical structures often have similar mechanisms of NO release. Most NO donors are low-molecular-weight compounds, including nitrates, nitrites, N-nitroso, C-nitroso, certain heterocycles, metal-NO complexes, and diazeniumdiolates [30]. Depending on the chemical nature of these compounds, NO is released spontaneously either in the presence or the absence of a catalyst [8]. 

Different classes of NO donors have been applied to studying biological systems. Seabra and Durán [31] described the use of disodium 1-[(2-carboxylato)pyrrolidin-1-yl]diazen-1-ium-1,2-diolate (PROLI/NO), 1-[N-(3-ammoniopropyl)-N-(n-propyl)amino]-diazen-1-ium-1,2-diolate (PAPA/NO), 1-[N-(3-aminopropyl)-N-(3-ammoniopropyl)diazen-1-ium-1,2-diolate (DPTA/NO) [32], 1-[N-(2-aminoethyl)-N-(2-ammonioethyl)amino]diazen-1-ium-1,2-diolate (DETA/NO) [33], S-nitrosoglutathione (GSNO) and S-nitroso-N-acetylcysteine (SNAC) [34–37], ruthenium derivatives [22, 38–40], and N-nitrosomelatonin (NOMela) [34]. However, according to Scatena et al. [13], while there are many new NO-releasing molecules, there are few real NO-releasing drugs. Among the molecules that are pharmacologically effective as NO-releasing drugs are organic nitrates (glycerol trinitrate, isosorbide dinitrate, isosorbide mononitrate, pentaerythritol tetranitrate, LA-419, piperazine derivative nitrates, and benzyl derivative nitrates), S-nitrosothiols (S-nitroso-N-acetylpenicillamine, S-nitroso-glutathione, S-nitroso-N-valerylpenicillamine, and S-nitroso-glucopyranose), diazeniumdiolates-NONOates (JS-K, CB-3-100, PABA/NO derivatives, and NONOate hybrid drugs (NONO-NSAIDs)), furoxans (CHF 2206, furoxans hybrid drugs), zeolites (mesoionic oxatriazoles (MOTA)), NO hybrid drugs (NO-hydrocortisone, NO-enalapril, and NO-ursodeoxycholic) and hydroxyurea.

NO donors can be incorporated into or chemically linked to biopolymers, mimicking endogenous NO production at a target site [31, 41]. Currently, the most critical obstacle to the development of new NO donor drugs is release at a specific tissue site at an optimal concentration, with the purpose of achieving a therapeutic effect and minimizing toxic effects [13].

### 1.3. NO and Nanotechnology

Although NO is used in many biomedical applications, its utility is limited by its short half-life, instability during storage, and potential toxicity. Efficient methods of both localized and systemic *in vivo* delivery and dose control are also lacking. Nanomaterials are currently being harnessed to overcome these limitations. These materials are usually able to load high amounts of NO, are quite stable, are sometimes photoactive, and possess demonstrable biological activity. Their surfaces can also be chemically modified and optimized for specific medical applications. 

There is particularly great interest in NO-releasing blood-compatible polymeric materials for coating medical devices, such as intravascular catheters, vascular grafts, coronary artery and vascular stents, and long-term vascular access devices. In these cardiovascular applications, continuous NO release over days and even months is desired [31]. Due to the crucial role of NO as an endogenous mediator of numerous physiological processes in the cardiovascular, immune, and nervous systems as well as in skin physiology, great effort has been devoted to the development of NO delivery systems for therapeutic purposes over the last few years [42].

Drug-delivery technologies are being widely used by pharmaceutical companies to expand the market for their already established products [43]. Over the past two decades, researchers have realized that nanotechnology is a fundamental part of drug development, resulting in the design of a wide range of drug-delivery systems [44, 45] and a progressive increase in the number of commercially available nanotechnology-based drugs [46–49]. Such novel delivery systems may reduce drug side effects, facilitate drug administration, ensure or improve patient compliance, decrease drug toxicity, enhance the bioavailability of drugs, and be tailored toward specific therapeutic targets [6, 43]. 

Nanotechnology is a relatively new area and its application in medicine is promising [45, 50, 51]. Nanoscale drug-delivery systems may increase the duration of drug circulation in the blood, allowing a reduction in the dose required to achieve therapeutic levels over an extended period of time. Nanomaterials may also deliver a drug directly to a target site, reducing its toxicity, which contributes to a decrease in side effects [52–55]. At this target site, nanosystems may accumulate at higher concentrations than conventional drugs due to their small size, potententially increasing the delivered drug's therapeutic efficacy [56]. Additionally, these delivery systems can be employed to target very inaccessible sites, such as the brain, if they are designed to permeate biological barriers [57]. Finally, the formulation of a drug in a nanoparticulate system can reduce renal and hepatic clearance and decrease immune system recognition, optimizing the drug's pharmacokinetic properties and biodistribution [58]. 

Nanocarriers not only improve drug solubility but also drug stability, allowing further development of potentially effective compounds that were rejected during preclinical or clinical research due to suboptimal pharmacokinetic or biochemical properties. Thus, nanocarriers may facilitate the development of multifunctional systems for targeted drug-delivery [59, 60], combined therapies [56, 61], or systems for simultaneous therapeutic and diagnostic applications. Nanocarriers of nitric oxide make the agent more available to the systemic circulation and also can enhance a target of NO, the interaction of nitric oxide with blood vessels, through of use of antibody moieties to selectively target drug-delivery vehicles to blood vessels. In the remainder of this paper, we will focus on the most clinically important NO-releasing nanostructures.

## 2. Polymeric Nanocarriers

### 2.1. Polymeric Nanoparticles and Micelles

NO is frequently administered via an NO donor, also known as a prodrug because of the difficulty of delivering it directly. However, most NO donors are labile, decomposing too rapidly to be useful, while the lifetime of NO itself in tissues is a mere 4–15 seconds, corresponding to a diffusion distance of approximately 150–500 *μ*m. The use of nanocarriers is one viable alternative for improving the stability and therapeutic delivery of NO [62, 63].

The use of polymeric nanoparticles and micelles as nanocarriers for drug delivery has been extensively investigated. These systems can be used to increase the aqueous solubility of drugs and to modulate drug activity by passive or active targeting to different tissues. Furthermore, biodegradable polymers can degrade into nontoxic monomers inside the body and are generally highly stable in biological fluids as well as during preparation and storage [64–66]. Such biodegradable and biocompatible polymers include polylactic acid (PLA), polyglycolic acid (PGA), and polylactic-co-glycolic acid (PLGA). The latter is approved for therapeutic use by the Food and Drug Administration (FDA) and is one of the most widely used polymers in nano- and microparticle production [31, 67]. 

**Figure 1:**
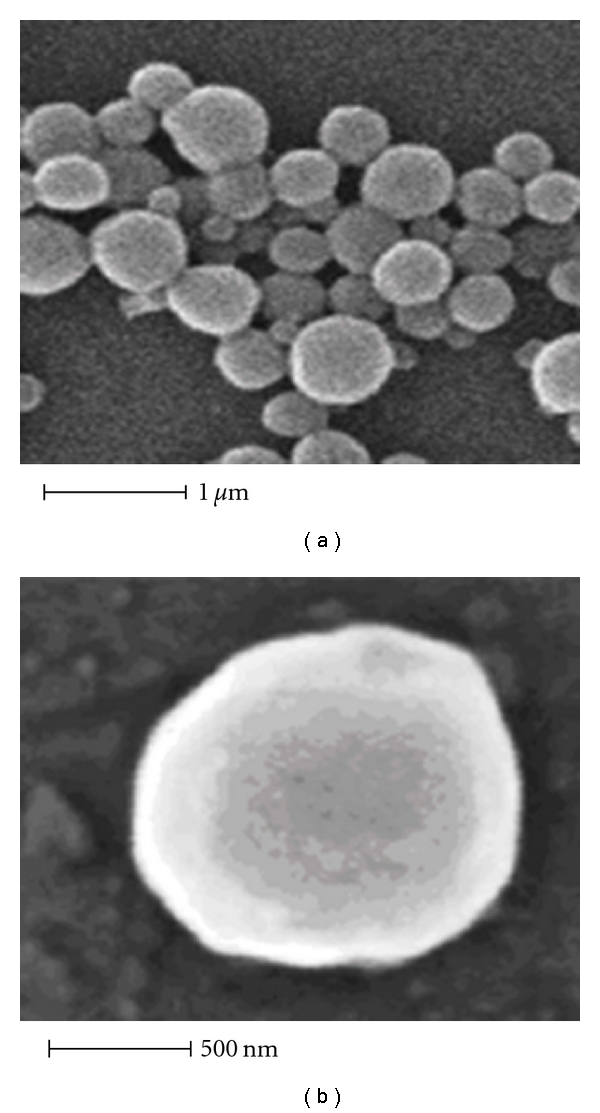
Scanning electron micrograph of polymeric nanoparticles containing the NO donor agent *trans*-[RuCl([15]ane)(NO)]^2+^. (a) Panoramic view. (b) Isolated magnified particle. Reprinted from Oliveira et al. [6], with the permission of Editorial Executive, Research Trends.

Polymeric particles with a diameter of less than 1 *μ*m [68] (Figure 1) have shown advantages over liposomes in physiochemical stability and encapsulation efficiency [69]. These nanoparticles can be prepared by physiochemical, chemical and mechanical methods [70]. However, drug release from particles may vary according to the polymer used or the drug encapsulated [71], while the method of encapsulation and the experimental conditions may influence particle size, morphology, and encapsulation efficiency [67].

Polymeric micelles are generally lower in size than nanoparticles and liposomes and larger than dendrimers, while sufficiently small (less than 100 nm in diameter) to penetrate tissues. Additionally, liposomes can be eventually dissembled after all drug has been delivered [63]. 

A small number of reports have been published on the delivery of NO using polymeric systems. Oliveira et al. [6] developed and characterized PLGA nanoparticles containing the NO donor agent (*trans*-[RuCl([15]ane)(NO)]^2+^). One year later, Jain et al. [71] demonstrated that stabilization of NO pro-drugs and anticancer lead compounds via their incorporation into polymer-protected nanoparticles composed of polystyrene-b-PEG (PS-b-PEG) and PLA-b-PEG may enhance their therapeutic effects. Meanwhile, Yoo et al. [72] described PLGA microparticles containing an NO donor that efficiently delivered NO to the vaginal mucosa, resulting in improved vaginal blood perfusion, which may have implications in the treatment of female sexual dysfunction. Another potential clinical application of polymeric nanocarriers is in topical NO delivery, such as by incorporation of NO donors into a liquid PEG/water matrix [31]. Finally, Kanayama et al. [66] reported that PEGylated polymer micelles may be capable of delivering exogenous NO to tumor cells in a photocontrolled manner, resulting in an NO-mediated antitumor effect, which indicates the promise of this polymeric system in NO-based tumor therapy.

### 2.2. Dendrimers and Hydrogels

**Figure 2:**
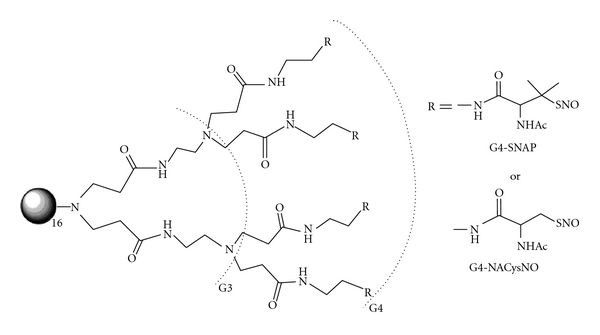
Generation-4 PAMAM with a completely modified exterior (64 thiols) of S-nitroso-N-acetyl-D,L-penicillamine (G4-SNAP) or S-nitroso-N-acetylcysteine (G4-NACysNO). Reprinted from Stasko et al. [76], with the permission of American Chemical Society, ACS Publications.

Dendrimers are monodisperse macromolecules with a tridimensional structure that is highly ramificated and regular around the nucleus [64, 73]. The ability to store NO on a dendritic scaffold using the NO donor N-diazeniumdiolate was first demonstrated by Stasko and Schoenfisch [74]. Benini et al. [75] then reported that the system formed by anchoring of K[RuIII(edta)(Cl)] to poly(amidoamine) dendrimers (PAMAM) can relax aortic rings lacking endothelium and exert trypanocidal effects. Meanwhile, Stasko et al. [76] synthesized two generation-4 PAMAM dendrimers with S-nitrosothiol exteriors (Figure 2) and characterized their ability to inhibit thrombin-mediated platelet aggregation. 

**Figure 3:**
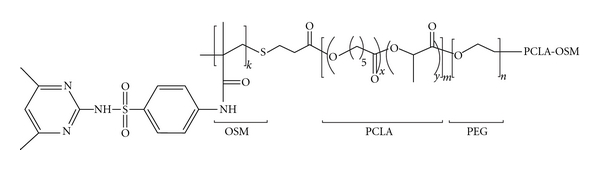
Structure of a OSM-PCLA-PEG-PCLA-OSM hydrogel. Reprinted from Nguyen and Lee [77], with the permission of Copyright and Licensing Manager, Wiley-VCH Verlag GmbH & Co.

Another interesting delivery system is hydrogel (Figure 3), a three-dimensional hydrophilic polymeric network that can absorb and retain a considerable amount of water while maintaining shape. This system has enormous potential in the design of closed-loop drug delivery. Due to their highly hydrophilic characteristics, biodegradable hydrogels have been widely used in biomedical applications, such as drug and cell delivery [64, 77]. 

The gradual release of NO from a material arises from a combination of the features of glassy matrices and hydrogels, as reported by Friedman et al. [78]. These researchers demonstrated that silane hydrogel containing NO promotes a reduction in angiogenesis, preventing bacterial dissemination from abscesses. Therefore, such materials may potentially serve as topically applied antimicrobials for the treatment of cutaneous infections and wounds [79, 80]. Additionally, NO-releasing hydrogel/glass hybrid nanoparticles may be preferable to other NO-releasing compounds because they do not depend on chemical decomposition, or enzymatic catalysis but rather only on the rate of hydration [23].

## 3. Lipid-Based Nanocarriers

### 3.1. Liposomes

Liposomes are vesicles formed by an aqueous core surrounded by one or several phospholipid bilayers. Hydrophilic drugs or active compounds can be incorporated into the inner aqueous cavity, while lipophilic drugs may be incorporated into the bilayer [81]. Liposomes are used as nanovehicles in various clinical applications and are potentially valuable vehicles for targeted therapeutics. One benefit of circulating liposomes is that they can accumulate in tissues with high vascular permeability by simple passive diffusion or extravasation, such as at the site of cancer, inflammation, or ischemia. Liposomes can also be surface-conjugated with molecules recognized by specific types of cells or tissues for targeted delivery [82]. 

Liposomes also have applications in molecular imaging, serving as a tool for diagnosis [83]. The encapsulation of gases into liposomal formulations results in a contrast agent that is suitable for ultrasound imaging. When this gas has a therapeutic application, such as NO, the resultant echogenic liposomes (ELIPs) can also be used for treatment of many diseases [84]. Huang et al. [84] specifically developed a bioactive gas-delivery method, using ELIPs as the NO carrier, to inhibit intimal hyperplasia. The release profile of NO from the NO-ELIP demonstrated an initial rapid burst followed by a more sustained release. The delivery of NO to VSMCs using the NO-ELIP was sevenfold greater than when unencapsulated NO was administered, and this liposome remained an effective delivery agent even in the presence of NO-binding hemoglobin. Furthermore, NO-ELIPs triggered a significant reduction in hyperplasia, in contrast with Ar-ELIPs [85].

Liposomes can be targeted to pathologic sites by conjugating antibodies and other ligands to the liposomes' phosphatidylethanolamine head groups [84]. This strategy could, for example, selectively target the drug to blood vessels, permitting more NO generating and also its accumulate in endothelial cells, promoting vasodilation. However, the direct conjugation of antibodies to NO-ELIPs results in greater than 90% loss of antibody immunoreactivity, mainly due to the denaturation induced by the gas pressurization and freeze-thawing procedures. To avoid this drawback, NO-ELIPs and antibodies have been linked by biotin/avidin-mediated coupling, providing a novel conjugation method allowing site-specific NO delivery [86].

**Figure 4:**
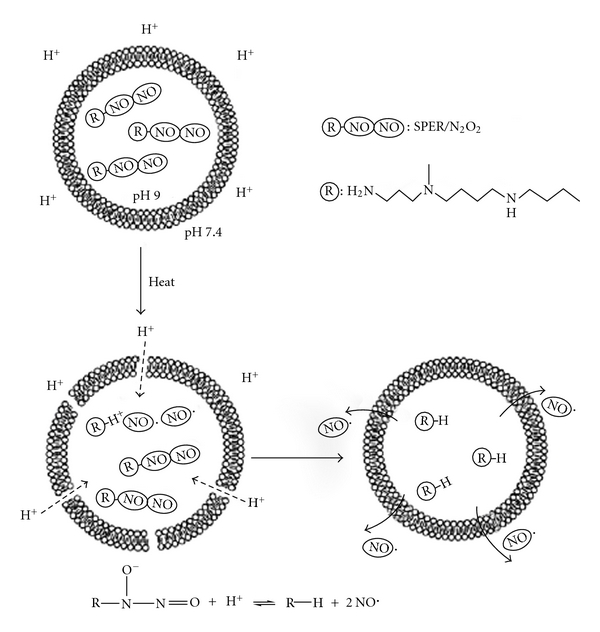
Schematic representation of the stabilization of zwitterionic diazeniumdiolate by loading liposomes. Reprinted from Tai et al. [89], with the permission of Elsevier.

Another class of liposomes that can be successfully used as nanocarriers is the thermosensitive liposomes, which may be employed in the storage, delivery, and active release of NO in a heat-mediated manner [87, 88]. Tai et al. [89] encapsulated spermine NONOate (SPER/N_2_O_2_), a zwitterionic diazeniumdiolate employed as an NO precursor, in liposomes composed of phospholipids of different temperature sensitivities (Figure 4). Upon heating, an influx of extraliposomal protons decreased the intraliposomal pH, diminishing the pH gradient across the membrane and subsequently inducing rapid NO release. The collapse of the pH gradient suggests that heat induced an increase in the lipids bilayer's permeability, allowing proton influx. SPER/N_2_O_2_ is known to spontaneously dissociate into two molecules at a much faster rate at physiological pH than at the basic pH, demonstrated by slower NO release from basic intra-liposomal solution than from physiological intra-liposomal solution. The degree of the slowed NO release was also dependent on the specific phospholipid composition of the liposomes. Moreover, the presence of a stronger pH gradient when the liposomes were applied to a more acidic environment increased proton influx and thus NO release. Because heat is generated in some pathological conditions, such as in tumor tissue, thermo-sensitive liposomes containing NO may have applications in anticancer therapeutics [8].

Dinh et al. [90] investigated the effect of the hydrophobic structure of liposomes' phospholipids and surfactant micelles on NO formation from zwitterionic diazeniumdiolates. The acid-catalyzed dissociation of NO has been examined in phosphate-buffered solutions of sodium dodecyl sulfate (SDS) micelles and 1,2-dipalmitoyl-*sn*-glycero-3-phosphocholine (DPPC) and 1,2-dipalmitoyl-*sn*-glycero-3-[phospho-(1-glycerol)] sodium salt (DDPG) phospholipid liposomes. Both phosphatidylcholine and phosphatidylglycerol liposomes catalyze NO dissociation from diazeniumdiolate substrates. The larger catalytic factors observed for DPPG liposomes than for DPPC liposomes and SDS micelles arise from the ability of the anionic liposome to concentrate the reactants at the liposome surface. This is accomplished through coulombic attraction of aqueous hydrogen ions and positive nitrogen centers in the diazeniumdiolate zwitterions. These findings provide insight into the interactions expected between diazeniumdiolate substrates and the charged aqueous interfaces that they may encounter when employed as NO donors in a biological environment.

Liposomes have also been used as models for bilayer membranes in studies to explore the effect of different phospholipids on diazeniumdiolate reactivity. It was found that anionic liposomes increased the dissociation rate of NO from diazeniumdiolate [91]. This study leads to a better understanding of the local environmental factors influencing NO donors' reactivity, and, since negatively charged phospholipids are important components of membranes and pulmonary surfactants, it may help explain the success obtained in experiments using diazeniumdiolate as a pulmonary vasodilator [92]. In another study, it was shown that positively charged liposomes, derived from the synthetic surfactant DOTAP, increase the dissociation of O^2^-arylated diazeniumdiolate prodrugs catalysed by the enzyme glutathionetransferase, in a membrane model system [91]. This prodrug has been successfully used to target NO to acute myeloid leukemia cells on activation by glutathione/glutathione *S*-transferase. [93–95]. A cationic liposome composed of DOTAP has been used to transfer the gene nitric oxide synthase (NOS) to vascular smooth cells, which indicates the potential therapeutic relevance for this transfer system to treat cardiovascular diseases [96]. These studies surely give insight into the use of charged liposomes as a strategy to deliver NO in a site-specific way, which would make them clinically more relevant. Cationic liposomes could be used, for example, not only for gene transfer, but to deliver a nitric oxide donor to blood vessels, which could enable a more potent vasodilatation because of the ability of cationic liposomes to interact with endothelium cells via electrostatic interaction [97].

### 3.2. Solid Lipid Nanoparticles

Solid lipid nanoparticles, composed of a lipid matrix stabilized by a surfactant, have great potential as drug-delivery systems due to their safety, high physical stability, and controlled release capability. Additionally, lipid carriers may enable the successful topical administration of many drugs due to attachment to the skin surface, allowing lipid exchange between the outermost layers of the carriers [98–100]. Solid lipid nanoparticles (SLNs) were first introduced in the early 1990s, followed by the second-generation technology of nanostructured lipid carriers (NLCs), particles produced using a blend of solid and liquid lipids to increase drug loading [101].

[Ru(Terpy)(bdqi)NO](PF_6_)3, an NO donor nitrosyl ruthenium complex (NRC), has been bound to lipid carriers for topical administration. This system exhibited improved stability in the skin and NO release by visible light irradiation, with potential applications in the treatment of skin cancer [102]. NO was constantly released in an NRC solution, in an SLN suspension and in an SLN solution in contact with skin, while its encapsulation was preserved when NRC-loaded SLNs were lyophilized. Additionally, photochemical studies demonstrated that ultraviolet-visible (UV-Vis) light irradiation triggered NO release from NRC-loaded SLNs approximately twofold greater than that of NRC in solution. This approach may be useful in improving NRC reduction.

## 4. Inorganic Nanocarriers

### 4.1. Gold Nanoparticles

Gold nanoparticles (GNPs) and gold monolayer-protected clusters (MPCs) provide attractive delivery vehicles [103, 104]. The gold core is inert and nontoxic, while the particles themselves are monodisperse and small in size (1.5–10 nm in diameter) and possess a dense gold core that allows imaging. Moreover, GNPs and MPCs exhibit unique chemical and physical properties defined by the protecting ligands used during their synthesis and their functional groups, such as thiols, phosphines, and amines, which exhibit an affinity for gold surfaces [105]. For example, GNP solubility is governed by the structure of the protecting ligand. The particles can be stabilized by a variety of thiol ligands that are readily modified by bromo-, amino-, and carboxy-terminated alkanethiols via place-exchange reactions. Using these functional groups, it is possible to anchor additional moieties, such as oligonucleotides, proteins and antibodies, generating multifunctional delivery systems for gene-based, antitumor, and antibacterial therapeutics [105]. 

Conventional methods of GNP synthesis consist of the reduction of aqueous tetrachloroaurate (AuCl_4_^−^) with sodium citrate or sodium borohydride [106, 107] and the Brust-Schiffrin method of two-phase synthesis and stabilization by thiols [108]. Several variations on these methods have also been implemented [109–111]. 

GNPs have been described as gene-delivery vehicles for the controlled and directed transport of plasmid DNA, siRNA or antisense oligonucleotides into living cells. Indeed, GNPs have arisen as a more effective alternative to viral vehicles, which can present unpredictable cytotoxicity and immune responses [103, 112]. GNPs functionalized with fluorescent ligands have been used to characterize interactions and mechanisms during various applications. Zhang et al. reported the fluorescence quenching of CdSe NPs by GNPs due to fluorescence resonance energy transfer (FRET), which can be utilized as a basis for ultrasensitive analytical techniques in biology and medicine [107].

GNPs may represent a novel platform for the targeted delivery of NO *in vivo* [113, 114]. GNPs functionalized with carboxy-terminated alkanethiols have been synthesized as a scaffold for NO-photoreleasing nanoparticles [31]. Meanwhile, Rothrock et al. reported the synthesis and stability of *N*-diazeniumdiolate, NO-donor GNPs modified with different amine-derivatized monolayers. Because NO is highly reactive and may disrupt gold-sulfur bonds, they studied the stability of these GNPs after exposure to high NO pressures. It was observed that NO release from diazeniumdiolate-modified GNPs was also tunable by varying the amine precursor structure, suggesting an NO release/diazeniumdiolate structure relationship. 

**Figure 5:**
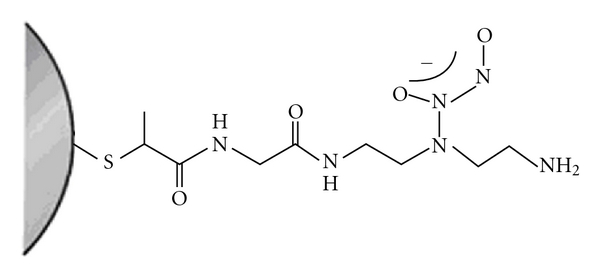
Nanocontainers for NO storage. Reprinted from Ghosh et al. [116], with the permission of Elsevier.

Further functionalization of GNPs with receptor molecules, enabling specific antibody-antigen or ligand-receptor interactions, may allow targeting to specific tissues or cells [114]. Such targeted controlled release of NO could be an effective therapy for hypoxic respiratory failure associated with pulmonary hypertension. Polizzi et al. demonstrated that NO can be efficiently stored by covalent linking to polyamine-stabilized GNPs via formation of acid labile N-diazeniumdiolate [115]. Additionally, they showed effective NO release from the water-soluble nanocontainers under acidic conditions (pH 3). pH-responsive materials have applications in drug delivery due to the mild acidic environment of inflamed tissues and tumors (pH ~  6.8) and in such cellular vesicles as endosomes (pH ~  5.5–6.0) and lysosomes (pH ~  4.5–5.0) (Figure 5) [116]. 

GNPs have been shown to catalyze NO generation whenever they come into contact with fresh blood serum. Meanwhile, NO has a relatively short lifetime in the blood due to its reactivity with various blood components, including hemoglobin. More abundant and stable forms of NO in the blood are *S*-nitroso adducts with thiol groups (RSNOs), such as *S*-nitrosoalbumin (AlbSNO), *S-*nitrosocysteine (CysNO), and *S*-nitrosoglutathione (GSNO) [117]. These compounds may function as NO-carrying systems, prolonging the half-life and spatial impact of NO. 

NO plays an important role in the control of vascular tone. It activates the soluble guanylyl-cyclase (sGC) and G-kinase protein, which decreases the cytosolic calcium concentration ([Ca^2+^]_c_) in the vascular smooth muscle cells (VSMCs) [118, 119]. GNPs have been synthesized and functionalized with nitrosyl ruthenium complex to investigate if this system potentiates the NO release and the vascular relaxation induced by the nitrosyl ruthenium complex. This NO-release GNP system induced a dose-dependent relaxation in endothelium denuded aortic rings better than the complex only [120].

### 4.2. Silica Nanoparticles

The physiochemical properties, stability and ability to form tunable porous structures with tailored surface functionalities has led to the possibility of using of silica nanoparticles (SiNPs) in the controlled delivery of drugs, biocides, genes, and proteins. Other advantages to SiNPs is that they are nontoxic and that their synthesis and isolation are straightforward [121]. SiNP nanoconjugation with NO donors is also advantageous [122], such as inorganic-organic hybrid SiNPs, functionalized ceramic materials prepared from silicon dioxide. The surface of such particles can be modified with reactive organic groups (amines, carboxylates, thiols, olefins, halides, and epoxides) capable of further functionalization using deliverable molecules or via free silanol groups.

**Figure 6:**
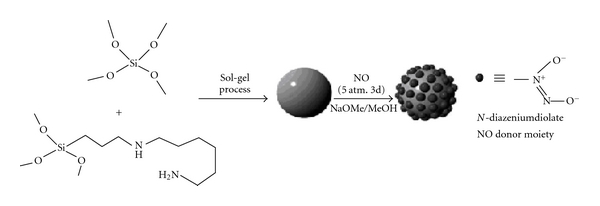
Schematic representation of the synthesis of *N*-diazeniumdiolate-modified SiNPs using TEOS and *N*-(6-aminohexyl)aminopropyltrimethoxysilane as tetraalkoxysilane and aminoalkoxysilane precursors. Reprinted from Seabra and Durán [31], with the permission of Royal Society of Chemistry (http://www.rsc.org).

A new synthetic approach to preparing NO-releasing SiNPs via a one-pot sol-gel process (Figure 6) includes cocondensation of tetraethoxysilane (TEOS) or tetramethoxysilane (TMOS) and aminoalkoxysilane with appropriate amounts of ethanol or methanol, water and ammonia. The amine functional groups within the SiNPs are subsequently converted into *N*-diazeniumdiolate NO donors via exposure to high NO pressures (5 atm) in the presence of sodium methoxide (NaOMe) base [31].

Das et al. [123] developed a novel method of controlled NO delivery to activated hepatic stellate cells (HSCs) in an *in vitro *setting resembling chronic liver disease. Several NO donors, such as S-nitroso-*N*-acetyl-DL-penicillamine (SNAP), glyco-SNAP, 3-morpholino-sydnonimine (SIN-1) and S-nitrosoglutathione (SNOG) were screened for long-term, slow NO-releasing ability and chemical characteristics. Au-SNAPs significantly attenuated the proliferation and vascular tube formation of the HSCs, an *in vitro *correlate of angiogenic phenotype, by releasing NO. Thus, the unique functionality of GNP- and SiNP-mediated drug-delivery systems may represent a new therapeutic approach to targeted NO delivery *in vivo *[123].

Stevens et al. engineered NO-releasing SiNPs for NO delivery to human ovarian cancer cells. They then compared the cytotoxicity of the SiNPs coupled to various ratios of an *N*-diazeniumdiolate in the presence of a small-molecule NO donor [PYRRO/NO: sodium 1-(pyrrolidin-1-yl)diazen-1-ium-1,2-diolate] to verify antitumor activity. This delivery system allowed control of the therapeutic payload, visualization of the nanoparticles via fluorescent tags, and exertion of NO-mediated anticancer effects [124].

*N*-diazeniumdiolates also have been employed to elucidate their potent effects on diverse NO-mediated disease states and pathophysiological disorders including cardiovascular disease and ischemia-reperfusion injury. However, the use of these compounds is limited due to their low solubility in physiological media, lack of specific targeting, and low capability to deliver therapeutic concentrations of NO, which decrease their potential clinical application. The coupling of the *N*-diazeniumdiolates to the nanoparticles delivery systems have been improved NO storage and release capability. Shin and Schoenfisch reported a new synthetic route to prepare NO-releasing silica particles through the methodology that permit the development of NO storage and delivery scaffolds for pharmacological applications [121].

### 4.3. Quantum Dots

**Figure 7:**
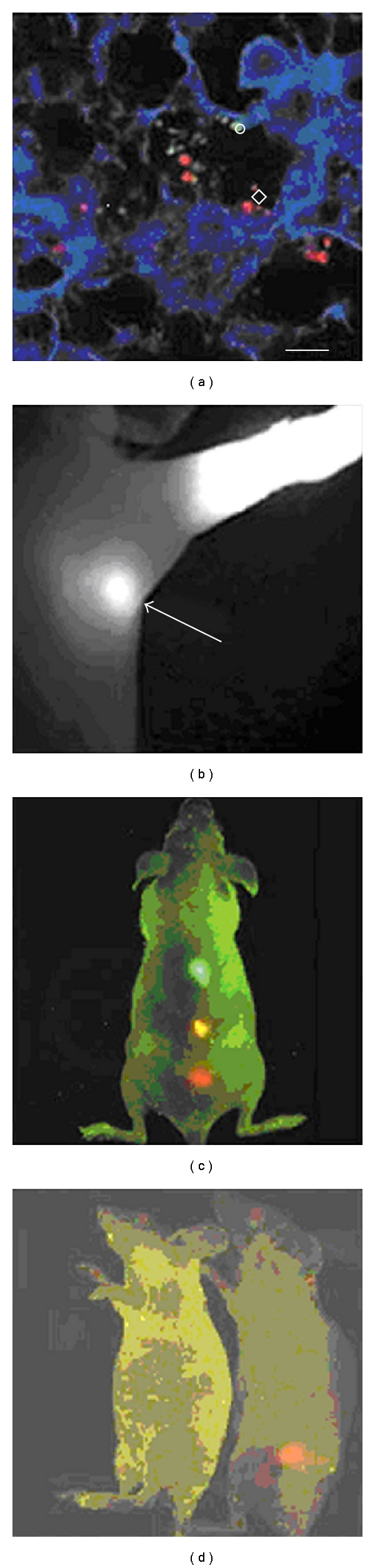
*In vivo* targeting and imaging using QDs. (a) *Ex vivo* tissue examination of QD-labeled cancer cells trapped in a mouse lung [129]. (b) Near-infrared fluorescence of water-soluble type II QDs taken up by sentinel lymph nodes [130]. (c) *In vivo* simultaneous imaging of multicolor QD-encoded microbeads injected into a live mouse [131]. (d) Molecular targeting and *in vivo* imaging of a prostate tumor in a mouse using a QD-antibody conjugate (red) [131]. Reprinted from Gao et al. [128], with the permission of Elsevier.

Nanotechnology can be exploited to improve the utility of fluorescent markers used for diagnostic purposes. Fluorescent nanocrystals, also known as quantum dots (QDs), may potentially overcome the inherent disadvantages of other common fluorescent markers, including the requirement of color-matched lasers, the occurrence of fluorescence bleaching and the lack of discrimination between multiple dyes. The bioimaging applications of QDs include *in vitro* and *in vivo* imaging of live cells and *in vivo* imaging of cancers and tumor vasculature [125, 126]. *In vivo* imaging using QDs has also been reported for lymph node mapping, blood pool imaging, and cell subtype isolation (Figures 7(a)–7(c)). Additionally, Ballou and coworkers [127] injected PEG-coated QDs into the mouse bloodstream and investigated how the surface coating affected circulation lifetime (Figure 7) [128].

QDs are formed as a core of semiconductor clusters of II–VI, III–V, and IV–VI column elements (as CdS, CdSe, CdTe, InAs, and GaN) with diameters of several nanometers. This core is usually covered by a surface-capping shell consisting of a passivating material that should be of a wider bandgap, or energy difference between the valence and conduction bands, than the core material, ZnS [132–135]. The presence of a shell dramatically increases the fluorescence quantum yields (QYs) of QDs nanocrystals by passivating surface nonradiative recombination sites [136] and also reduces leaching of damaging metal ions by oxidation from the surface [134, 135]. 

Typically, QDs are synthesized in organic solvents and exhibit hydrophobic surface ligands that could be replaced by such water-soluble bifunctional molecules as peptides, antibodies or nucleic acids [137–144]. For biological applications, the CdSe/ZnS core/shell composite is the best available QD fluorophore because its chemistry is the most refined [145]. 

**Figure 8:**
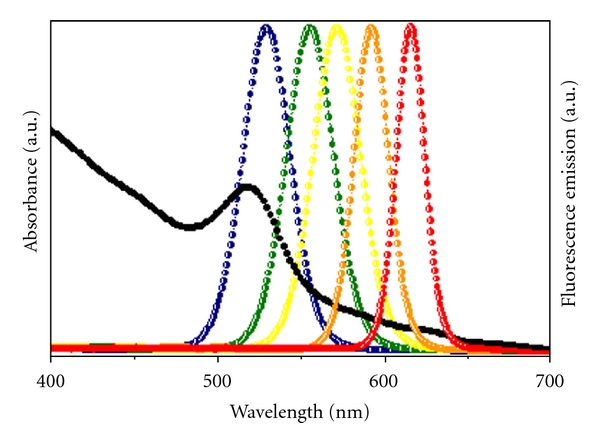
Size-dependent optical effects of semiconductor nanoparticles. Semiconductor nanoparticles contain size-dependent electronic and optical properties. A series of five different emission spectra of sized ZnS-capped CdSe nanoparticles called QDs is used to demonstrate this principle (colored dotted lines), in juxtaposition with the absorption spectrum (solid black line).

QDs exhibit a broad absorption spectrum for single excitation sources; a large molar absorption coefficient that increases toward the UV region; a narrow and symmetric emission spectrum for multiple-color imaging (full width at half maximum *<*30–40 nm), a high-fluorescence QY, and superior photostability [146]. The onset of absorption and the spectral position of the emission band (Figure 8) can be easily tuned by controlling the particle size and their material composition [132]. These unique optical and electronic properties justify the increasing research into and application of QDs in imaging of cellular cancer targets, *in vivo* multiphoton fluorescence for deep tissue visualization, and FRET- based sensing [134, 135, 147].

One of the most important emerging applications of QDs is traceable drug delivery [148], which has the potential to elucidate the pharmacokinetics and pharmacodynamics of drug candidates and to provide design principles for drug carrier engineering. For nanocarrier development and optimization, QDs can serve as an excellent prototype from which biocompatible carriers of similar sizes and surface properties can be made for clinical uses. Current applications of QDs in drug delivery are focused on two major areas: using QDs as carriers and labeling therapeutics [149] or coupling drug carriers with QDs [149, 150]. 

The investigation of luminescence nanoparticles as light sources for cancer therapy is also very interesting. The intense and stable emission fluorescence, high QY, large molar absorption coefficient in a wide spectral range, and the ability to transfer energy of QDs permit their use as photosensitizers in photodynamic therapy (PDT). Recent research has focused on developing photosensitizing QDs for the production of radicals upon absorption of visible light. In spite of the fact that visible light is safe, this approach is only suitable for the treatment of superficial tumors [151]. 

Cancer treatment requires high accuracy in delivering ionizing radiation to reduce toxicity to surrounding tissues. In the QD structure, multiple surface ligand sites provide the opportunity to tether functional groups to the surface, improving solubility properties and biological specificity [152]. 

The energy transfer between QDs and molecules in cells (such as triplet oxygen (^3^O_2_)) can induce the generation of reactive oxygen species (ROS) in the form of singlet oxygen (^1^O_2_) and anion superoxide (O_2_^−^), which promote apoptosis [22]. Intracellular release of QDs can be facilitated by functionalization, resulting in soluble, biocompatible QDs. QDs linked to NO-donor molecules can specifically lead to effective treatment of large tumors by PDT [153]. In this case, the nitrosyl compounds can generate, under light application, ROS and nitrogen (NOS) species via QD excitation, enabling tumor cell death [22, 152].

Neuman et al. [152] demonstrated enhanced NO photogeneration in *trans*-Cr(cyclam)(ONO)^2+^ (cyclam = 1,4,8,11-tetraazacyclotetradecane) when conjugated to water-soluble CdSe/ZnS core/shell QDs, indicating that the QDs may sensitize photoreactions of this nitrite complex. Numerous papers have related the use of nitrosyl or nitrite compounds that release NO under visible light irradiation in PDT. Furthermore, some of these compounds can also be applied as vasodilators, delivering NO in response to reductor stimuli [19, 153].

## 5. Innovations and Intellectual Property

The storage of NO and its controlled release from donors is difficult, partly due to the gaseous nature of NO and its instability in the presence of oxygen. Therefore, effective NO delivery systems are highly desirable and potentially lucrative, leading to the patenting of several inventions that combine NO donors with nanotechnology. Liposomes, capable of delivering one or more NO generators when composed of dimyristoylphosphatidylcholine (DMPC) and dimyristoyl-phosphatidylglycerol (DMPG) [154], were intellectually protected. Another invention described liposome formation from lipids containing the S-nitroso moiety –S–N]O, the O-nitroso moiety –O–N]O and/or an N-nitroso moiety, including the NONOates, resulting in beneficial therapeutic effects [155, 156]. 

NO-releasing nanomaterials have also been protected by patents, including systems based on carbon nanotubes. These nanomaterials contain NO or gases with NO-like biological activity, with the gases noncovalently bound to a compound, allowing both the storage and the controlled release of NO gas. Compounds disclosed in the invention include polymers, articles, pills, capsules, and medical devices [157].

Polymeric micelles for the delivery of NO have been patented, such as micelles for N-diazeniumdiolate administration [158]. Nano- and microparticulates for NO release have also been legally protected. One such invention provides an oral therapeutic comprising at least one NO donor coupled with an orally acceptable carrier [159]. Another patent describes the synthesis of biodegradable and nonbiodegradable nanoparticles for coating medical devices, such as intracoronary stents, in order to deliver NO donors and other active drugs [160]. Nanoparticulate systems containing a metallic cluster core (gold, platinum, silver, magnetite, quantum dots, or a combination thereof), a dendritic network core (polypropylenimine, polypeptide, polyamidoamine, polyarilether, polyesther, polyamide, triazine dendrimer, or dendritic polyglycerol), a cocondensed silica network, or a combination thereof have also been patented [161]. Finally, dendrimers for NO delivery are protected by patent [162]. 

Despite considerable advances and numerous patents, there are currently no commercially available nano- or microcarriers for NO delivery.

## 6. Considerations

The clinical potential of NO-containing particles is significant, although several prerequisites are necessary, including optimized delivery strategy, tissue targeting, and controlled and sustained NO release. Current nanotechnology-based systems are highly promising with respect to these properties. The extended circulation of particles with concomitant systemic delivery of NO could be used to treat several disorders such as systemic infections and malignant hypertension. Nanotechnology may also prove useful in the local delivery of NO to treat peripheral vascular disease, chronic wounds, and other conditions associated with endothelial dysfunction and poor perfusion. Nanotechnology may also prove useful in the local delivery of NO to treat peripheral vascular disease, chronic wounds and other. Nanocarriers of nitric oxide make the agent more available to the systemic circulation and also can enhance a target of NO, the interaction of nitric oxide with blood vessels, through of use of antibody moieties to selectively target drug-delivery vehicles to blood vessels. However, at present, there are many new NO-releasing molecules but few effective NO-releasing drugs.
